# Recent Advances in Design and Synthesis of Diselenafulvenes, Tetraselenafulvalenes, and Their Tellurium Analogs and Application for Materials Sciences

**DOI:** 10.3390/molecules27175613

**Published:** 2022-08-31

**Authors:** Nataliya A. Makhaeva, Svetlana V. Amosova, Vladimir A. Potapov

**Affiliations:** A.E. Favorsky Irkutsk Institute of Chemistry, Siberian Division of The Russian Academy of Sciences, Favorsky Str., Irkutsk 664033, Russia

**Keywords:** diselenafulvenes, tetraselenafulvalenes, ditellurafulvenes, tetratellurafulvalenes, heterocycles, materials sciences, superconductors

## Abstract

The first organic metals were obtained based on tetrathiafulvalene. The most significant advance in the field of organic metals was the discovery of superconductivity. The first organic superconductors were obtained based on tetramethyltetraselenafulvalene. These facts demonstrate great importance of tetraselenafulvalenes and their precursors, diselenafulvenes, for materials sciences. Derivatives of 1,4-diselenafulvene and 1,4,5,8-tetraselenafulvalene are useful building blocks for organic synthesis and donor units for the preparation of charge-transfer complexes and radical ion salts, the construction of organic metals, superconductors, organic Dirac materials, semiconductors, ferromagnets, and other conductive materials. This review covers the literature on the design, synthesis, and application of 1,4,5,8-tetraselenafulvalenes and 1,4-diselenafulvenes and their tellurium analogs over the past 15–20 years. These two classes of compounds are interconnected, since the main part of methods for the synthesis of tetraselenafulvalenes is based on the diselenafulvene derivatives as starting compounds. Special attention is paid to the development of novel efficient synthetic approaches to these classes of compounds. Conducting properties and distinguishing features of materials based on tetraselenafulvalenes and their tellurium analogs as well as examples of materials with high conductivity are discussed.

## 1. Introduction

Historically, the first organic metals based on 1,4,5,8-tetrathiafulvalene (TTF) were discovered. The preparation of a conducting TTF salt was described in 1972 [1] and the synthesis of first organic metal, a complex of TTF with tetracyanoquinodimethane (TCNQ), was reported in 1973 [2]. The TTF-TCNQ complex behaved as a metal over a large temperature range and had by far the largest maximum electrical conductivity of any organic compound known at that time [2].

The discovery of organic metals gave impetus to the development of synthetic approaches to a variety of structural modifications of TTF, which have been carefully studied in search of organic metals with high conductivity [3,4,5]. As the replacement of skeletal sulfur atoms of TTF by more polarizable selenium atoms has been generally recognized as an effective approach to superior electron donors with enhanced intermolecular interactions, selenium analogs of TTF, 1,4,5,8-tetraselenafulvalene (TSF), have received much attention and have been intensively studied as electron donors for the preparation of conducting materials [4,5,6].

The most significant advance in the field of organic metals has been the discovery of their superconductivity. The first organic superconductor was synthesized by Bechgaard et al. based on tetramethyltetraselenafulvalene (TMTSF) [6]. It was obtained in the form of single-crystal salts with a composition of 2:1, (TMTSF)_2_PF_6_, by electrochemical oxidation of TMTSF in the presence of the corresponding tetraalkyl ammonium salts. Later, a number of similar salts, which exhibit superconductivity, were synthesized based on TMTSF and were named Bechgaard’s salts [6]. These historical facts demonstrate great importance of TSF derivatives and their precursors, 1,4-diselenafulvene, for materials sciences.

The Bechgaard’s salts have the general formula (TMTSF)_2_X, where X^−^ is a monovalent anion. The formation of the charge transfer salts includes the transfer of one electron from two TMTSF molecules to one X molecule. 

Derivatives of 1,4-diselenafulvene and 1,4,5,8-tetraselenafulvalene are of great interest as important heterocyclic building blocks and scaffolds for organic synthesis. These two classes of compounds are interconnected, since the methods for the synthesis of tetraselenafulvalenes are considerably based on diselenafulvene derivatives. The tetraselenafulvalene derivatives serve as donor units for the preparation of charge-transfer complexes and radical ion salts, the construction of organic metals [7,8,9,10,11], semiconductors [12,13,14], superconductors [15,16,17,18,19,20,21,22], ferromagnets [23], and other conducting materials [24,25,26,27,28,29,30,31,32]. It should be noted that the bis(ethylenedithio)tetraselenafulvalene (BETS) derivatives yielded more organic metals and superconductors than other TSF derivatives [20,21,22]. The unsymmetrical analog of BETS, bis(ethylendithio)dithiadiselenafulvalene (BEDT-STF), is also an important donor. Various new molecular conductors including organic Dirac materials and magnetic conductors have been developed based on the BETS and BEDT-STF salts [20,21,22,31,32].

In 2004, the journal “Chemical Reviews” published an issue devoted to the preparation and properties of organic conductors and superconductors, as well as methods for the synthesis of their molecular components, in which significant place was given to the TSF derivatives [28,29,30]. The tellurium analogs, 1,4-ditellurafulvene and 1,4,5,8-tetratellurafulvalene derivatives, are also of high interest, as it can be seen from a 2003 review [33], which is devoted to the synthesis and physicochemical properties of 1,4-dichalcogenafulvenes and 1,4,5,8-tetrachalcogenafulvalenes.

However, since then and to the present, a number of novel synthetic approaches to 1,4-diselenafulvene and TSF derivatives have been developed and new data for material sciences have been obtained that require processing and rationalization. This article presents a review of the literature on 1,4-diselenafulvene and TSF derivatives, as well as on tellurium analogs of these compounds, mainly for the last 15–20 years. 

## 2. The 1,4-Diselenafulvene and 1,4,5,8-Tetraselenafulvalene Derivatives

### 2.1. Non-Condensed 1,4-Diselenafulvenes

**Scheme 1:**
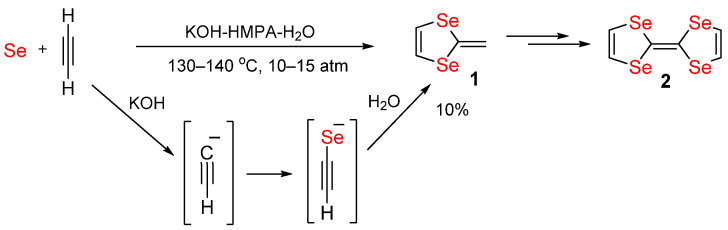
Synthesis of 1,4-diselenafulvene **1** from acetylene and selenium [34].

1,4-Diselenafulvene **1** was obtained based on available and inexpensive industrial starting reagents, selenium and acetylene, in the KOH-HMPTA-H_2_O system (100–140 °C, 10–15 atm). The proposed route for the formation of compound **1** includes the generation of the acetylide anion from acetylene and potassium hydroxide, its insertion reaction with selenium, followed by heterocyclization of the resulting ethyneselenolate anions (Scheme 1) [34].

**Scheme 2:**
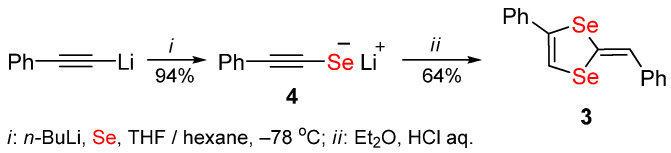
The preparation of (*E*)-2-benzylidene-4-phenyl-1,3-diselenole **3** from phenylacetylene and selenium [38,39].

Heterocycle **1** is an important intermediate in organic synthesis as well as the starting material for preparation of 1,4,5,8-tetraselenafulvalene **2**. The latter is the electron donor for the synthesis of organic metals: charge transfer complexes and radical ion salts with high electrical conductivity.

(*E*)-2-Benzylidene-4-phenyl-1,3-diselenole **3** is a promising product, which exhibits antioxidant, hepatoprotective, and anticonvulsant activity [35,36,37]. Protonation of lithium phenylalkyneselenolate **4** leads to dimerization and the formation of 1,3-diselenole **3** in 64% yield. Phenylacetylene was deprotonated with *n*-BuLi in THF solution at low temperature (−78 °C) in order to obtain lithium phenylalkyneselenolate **4**. This lithium derivative was used in situ in the insertion reaction with elemental selenium producing compound **4** in 94% yield (Scheme 2) [38,39].

Diselenole **3** was previously obtained in 94% yield by the one-pot efficient method based on the reaction of phenylacetylene with elemental selenium in the system KOH-HMPA-H_2_O [40].

**Scheme 3:**
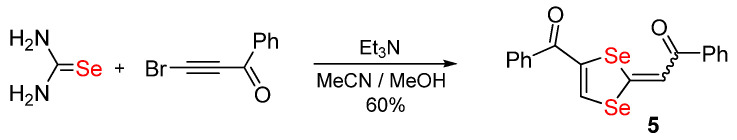
The synthesis of 3,5-dibenzoyl-1,4-diselenafulvene (**5**) by the reaction of selenourea with benzoylbromoacetylene in the presence of triethylamine [41].

Selenoketones are promising intermediates for the synthesis of selenium-containing heterocycles [41,42,43]. The reaction of selenourea with benzoylbromoacetylene in the presence of triethylamine led to the formation of 3,5-dibenzoyl-1,4-diselenafulvene (**5**) in 60% yield (Scheme 3) [41].

**Scheme 4:**
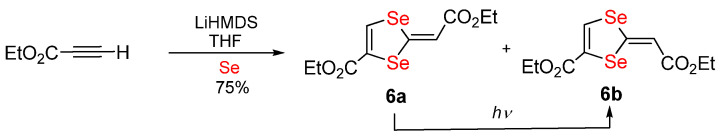
The preparation of ethyl 2-(2-ethoxy-2-oxoethylidene)-1,3-diselenole-4-carboxylate as a mixture of *E-* and *Z-*isomers (**6a**,**b**) [44].

Lithiation of ethyl propiolate with lithium hexamethyldisilazide in THF followed by selenium insertion reaction gives ethyl 2-(2-ethoxy-2-oxoethylidene)-1,3-diselenole-4-carboxylate in 75% yield as a mixture of *E-* and *Z*-isomers (**6a**,**b**) (Scheme 4) [44].

The recrystallization of this mixture made it possible to obtain pure *Z*-isomer **6b** suitable for X-ray diffraction analysis. Diselenoles **6a**,**b** turned out to be interesting crystalline compounds that exhibit an unusual coordination between the oxygen atom of the oxoethylidene group and the nearest selenium atom [44]. It is interesting to note that, under the action of the daylight, the *E*-isomer **6a** in solution was completely converted to the *Z*-isomer **6b** (Scheme 4), presumably through a photochemically induced isomerization mechanism. This conversion did not occur in the dark [44].

**Scheme 5:**
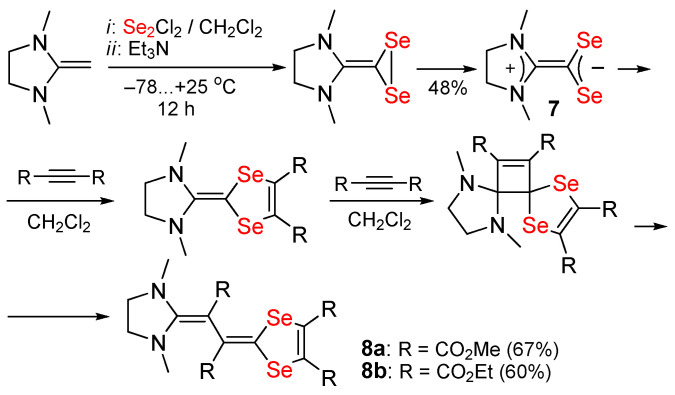
The synthetic route to compounds **8a**,**b** based on 2-methylene-1,3-dimethylimidazolidine and selenium monochloride [45].

The reaction of 2-methylene-1,3-dimethylimidazolidine with selenium monochloride followed by treatment with triethylamine gave 2-(1,3-dimethylimidazolidinium) diselenocarboxylate (**7**) in 48% yield as thermo stable crystals. This compound reacted with two equivalents of dimethyl- and diethyl acetylenedicarboxylates in dichloromethane at room temperature to afford 1:2 adducts **8a**,**b** in 67% and 60% yields, respectively (Scheme 5) [45].

**Scheme 6:**
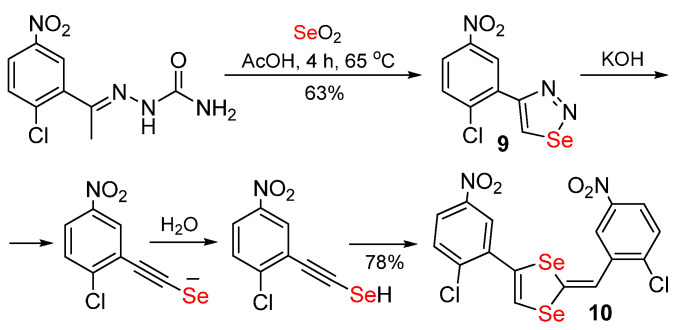
The new convenient approach to (*Z*)-2-(2-chloro-5-nitrobenzylidene)-4-(2-chloro-5-nitrophenyl)-1,3-diselenole **10 [46]**.

The new convenient approach to (*Z*)-2-(2-chloro-5-nitrobenzylidene)-4-(2-chloro-5-nitrophenyl)-1,3-diselenole **10** in 78% yield was developed based on available 4-(2-chloro-5-nitrophenyl)-1,2,3-selenadiazole **9** (Scheme 6). The nature and position of the substituents in compound **10** make it convenient for further use as a building block for obtaining more complex derivatives. For example, the nitro group in the aryl ring can be reduced to the amino group, while the halogen atom can be used in various cross-coupling reactions [46].

**Scheme 7:**
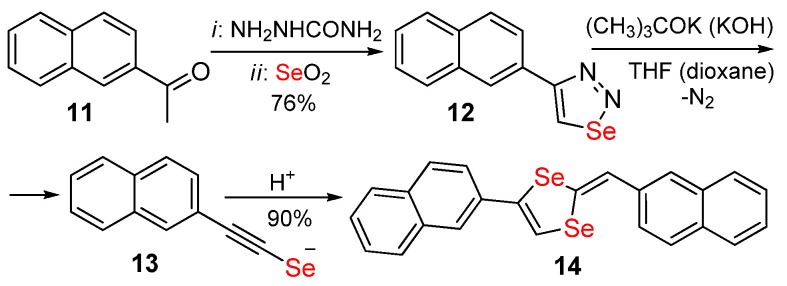
Synthesis of 4-(2-naphthyl)-2-[1-(2-naphthyl)methylidene]-1,3-diselenole **14** from 2-naphthylmethyl ketone **11**, selenium dioxide, and semicarbazide [47,48].

4-(2-Naphthyl)-1,2,3-selenadiazole **12** was obtained in 76% yield from 2-naphthylmethyl ketone **11**, selenium dioxide, and semicarbazide. Upon treatment with potassium tert-butylate in anhydrous THF or potassium hydroxide in absolute dioxane, selenadiazole **12** was converted to 2-(2-naphthyl)potassium ethyneselenolate **13**, which, in turn, underwent dimerization to 4-(2-naphthyl)-2-[1-(2-naphthyl)methylidene]-1,3-diselenole **14** in 90% yield upon protonation (Scheme 7) [47,48].

Diselenafulvene **14** was also obtained by treating a dioxane solution of compound **12** with an ethanolic potassium hydroxide solution [47,48].

### 2.2. Condensed 1,4-Diselenafulvenes and 1,4,5,8-Dithiadiselenafulvalenes

**Scheme 8:**
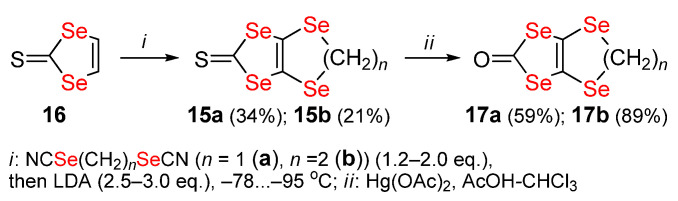
The synthesis of 1,3-diselenole-2-thiones **15a**,**b** and methylene- and ethylenediselenole derivatives **17a**,**b** [49].

A new method for the synthesis of 4,5-alkylene-diseleno-1,3-diselenole-2-thiones **15a**,**b** was developed without using the highly toxic reagent carbon diselenide (CSe_2_). The reaction of lithiated thione **16** with bis(selenocyanato)methane or 1,2-bis(selenocyanato)ethane was carried out at low temperature in dry THF leading to products **15a**,**b**. The latter compounds were readily converted to the corresponding ketones **17a**,**b** in 59% and 89% yields, respectively, by the conventional method using Hg(OAc)_2_/acetic acid/chloroform (Scheme 8) [49].

Methylene- and ethylenediselenole derivatives **17a**,**b** are valuable precursors for the preparation of a wide range of tetraselenafulvalenes.

**Scheme 9:**
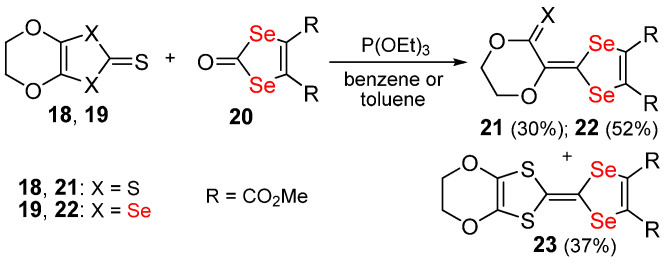
The synthesis of compounds **21**, **22**, and **23 [50]**.

It was found that the cross-coupling of 4,5-ethylenedioxy-1,3-dithiol- and -1,3-diselenole-2-thione (**18** and **19**) with compound **20** in the presence of triethylphosphite in refluxing toluene or benzene proceeded with abnormal ring opening giving 2-(thioxomethylidene)- and 2-(selenoxomethylidene)-1,3-diselenoles (**21** and **22**) in 30% and 52% yields, respectively (Scheme 9) [50]. In the reaction of thione **18** with ketone **20**, along with compound **21**, the expected product, ethylenedioxy-1,3-diselene-1,3-dithiafulvalene (**23**) in 37% yield was formed, while the reaction of thione **19** with ketone **20** proceeded without the formation of the corresponding tetraselenafulvalene (Scheme 9). The proposed pathway for the formation of heterocycles **21** and **22** included the attack of triethylphosphite on the thiocarbonyl group of compounds **18** and **19**, which promoted the breaking of neighboring C-S and C-Se bonds, followed by cross-coupling of the formed intermediates with ketone **20 [50]**.

**Scheme 10:**
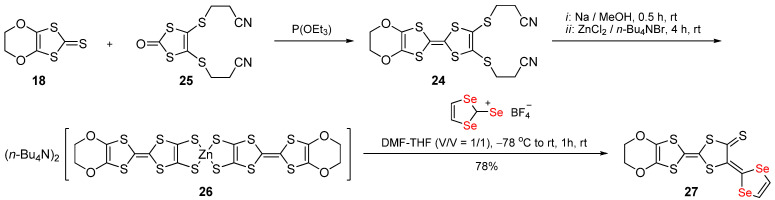
The synthesis of diselenafulvene **27**, an electron donor [51].

New functionalized diselenafulvene **27** was synthesized in 78% yield based on 1,3-diselenole-2-selone and zinc complex of intermediate compound, cyanoethylsulfanyl-substituted ethylenedioxytetrathiafulvalene **24**, which was obtained by cross-coupling of 4,5-ethylenedioxy-1,3-dithiol-2-thione **18** with 4,5-bis(2-cyanoethylthio)-1,3-dithiol-2-one (**25**) in the presence of triethylphosphite (Scheme 10) [51].

Studies of the electrochemical properties of the compound **27** showed that it is a good electron donor, which can be used to obtain conducting materials [51].

**Scheme 11:**
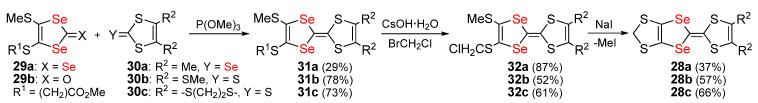
The synthetic approach to derivatives of 2,3-cyclohexylenedithio-1,4-dithia-5,8-diselanafulvalene [52].

The synthesis of dimethyl-, bis(methylthio)-, and ethylenedithio derivatives of dithiadiselenafulvalene **28a**–**c** is outlined in Scheme 11 [52]. The first of these three-step pathways was a cross-coupling reaction between 4-methylthio-5-(2-methoxycarbonylethylthio)-1,3-diselenole-2-selone or -1,3-diselenole-2-one **29a**,**b** with 1,3-dithiole-2-chalcogenones **30a**–**c** in the presence of trimethylphosphite. Yields of cross-coupling products **31a**–**c** were 29%, 78%, and 73%, respectively. At the second stage, compounds **31a**–**c** were subjected to deprotection of the 2-methoxycarbonylethylthio group with cesium hydroxide in a DMF solution and in situ treatment with bromochloromethane to give the corresponding 2-methylthio-3-chloromethylthio derivatives of dithiadiselenafulvalene **32a**–**c** in 87%, 52%, and 61% yields, respectively. The ring closing reaction via transalkylation at the sulfur atom was initiated by treatment with sodium iodide to obtain derivatives **28a**–**c** in 37%, 57%, and 66% yields, respectively. The resulting compounds were found to have good electron-donating properties. A radical cation salt with AsF_6_^−^ anion, which exhibited semiconductor properties, was obtained based on compound **28c [52]**. 

**Scheme 12:**
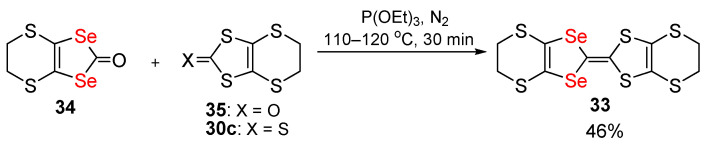
The synthesis of bis(ethylenedithio)diselenadithiafulvalene **33** [53].

One of the most important donors, unsymmetrical bis(ethylenedithio)diselenadithiafulvalene (BEDT-STF) **33**, can be obtained by the method depicted in Scheme 12 [53]. The cross-coupling reaction of 4,5-ethylenedithio-1,3-diselenole-2-one **34** with 4,5-ethylenedithio-1,3-dithiol-2-thione **30c** proceeded in triethylphosphite at 110–120 °C for 30 min in a nitrogen atmosphere affording product **33** in 46% yield. The yield of product **33** was significantly lower (7%) in the case of using 4,5-ethylenedithio-1,3-dithiol-2-one **35** (Scheme 12) [53].

While most organic compounds require high pressures to exhibit Dirac-cone-type band structures, the organic charge-transfer complex (**33**)_2_I_3_ exhibits unique properties to form Dirac electron states under ambient pressure [32,53,54,55,56,57]. 

**Scheme 13:**
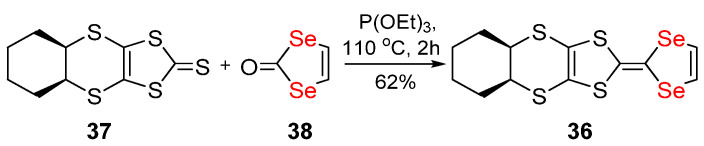
The efficient synthesis of 2,3-cyclohexylenedithio-1,4-dithia-5,8-diselanafulvalene **36** [58].

The efficient synthesis of 2,3-cyclohexylenedithio-1,4-dithia-5,8-diselanafulvalene **36** was developed based on the cross-coupling reaction of 4,5-cyclohexylenedithio-1,3-dithiole-2-thione **37** with 1,3-diselenole-2-ketone **38** (Scheme 13) [58]. The reagents were heated at 110 °C in the presence of triethylphosphite for 2 h. After cooling the mixture to room temperature, removing the solvent, and the purification by column chromatography, the target product **36** was obtained in 62% yield. 

The product **36** revealed donor properties and was used for the preparation of a new maleonitrile dithiolate nickel complex, **36**·Ni[S(CN)C=C(CN)S]_2_. It was found, however, that this complex had usual mixed donor-acceptor stacks and exhibited dielectric properties [58].

**Scheme 14:**
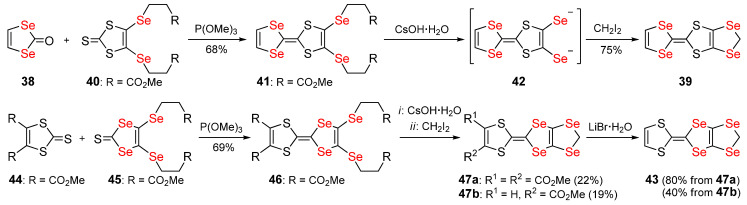
The synthetic routes to 5-*H*-(1,3-diselenole-2-ylidene)-1,3-dithia-4,6-diselenapentalene **39** and 5-*H*-(1,3-dithiol-2-ylidene)-1,3,4,6-tetraselenapentalene **43** [59].

The synthetic approach to 5-*H*-(1,3-diselenole-2-ylidene)-1,3-dithia-4,6-diselenapentalene **39** is shown in Scheme 14 [59]. The cross-coupling reaction of 1,3-diselenole-2-one **38** and 4,5-bis[(2-methoxycarbonyl)ethylseleno]-1,3-dithiol-2-thione **40** gave 2,3-bis[(2-methoxycarbonyl)ethylseleno]diselenadithiafulvalene **41** in 68% yield. Then, deprotection of the methyl propionate group in compound **41** with cesium hydroxide led to the formation of diselenadithiafulvalene diselenolate dianione **42**, which was realkylated with diiodomethane to afford the expected product, methylene diselenadithiadiselenafulvene **39** in 75% yield (Scheme 14) [59].

The preparation of 5-*H*-(1,3-dithiol-2-ylidene)-1,3,4,6-tetraselenapentalene **43** was carried out according to a modified procedure using 4,5-bis(methoxycarbonyl)-1,3-dithiol-2-thione **44** instead of the original 1,3-dithiol-2-thione **35** (Scheme 14) [59].

The cross-coupling of thione **44** with compound **45** gave the desired unsymmetrical intermediate product **46** in 69% yield. The formation of the selenium-containing ring was carried out in the same way as for the synthesis of compound **39**. However, in this case, a mixture of the reaction products, diester **47a** and monoester **47b**, was formed. The latter compound was a partially deesterified product, the formation of which was probably caused by the action of cesium iodide. Precursors **47a** and **47b** were converted to product **43** using standard deesterification conditions (Scheme 14) [59].

The compounds **39** and **43** exhibited fairly good electron-donor properties among the selenium-modified series of methylenedithio tetrathiafulvalenes. Based on these new donors, electrocrystallization in the presence of tetrabutylammonium salts (*n*-Bu_4_NX, X = Br, AuI_2_, I_3_) gave radical-cationic salts *κ*-(**39**)_2_Br, *θ*-(**39**)I_1.26_, (**43**)_2_(AuI_2_)_0.44_, and (**43**)I_1.26_. All the obtained salts showed metallic properties down to a temperature of 1.5 K [59].

**Scheme 15:**
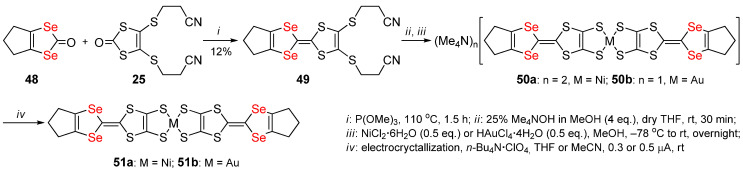
The synthesis of trimethylenediselenadithiafulvalene **49** and its complexes with nickel **50a**,**b** and gold **51a**,**b [59]**.

The cross-coupling reaction of 4,5-trimethylene-1,3-diselenole-2-one **48** with 4,5-bis(2-cyanoethylthio)-1,3-dithiol-2-one **25** in the presence of trimethyl phosphite at 110 °C gave trimethylenediselenadithiafulvalene **49** in 12% yield. The diselenadithiafulvalene **49** was used in the synthesis of components of single metal complexes. Further deprotection of compound **49** and its treatment with a methanol solution of NiCl_2_∙6H_2_O or HAuCl_4_∙4H_2_O in the temperature range from −78 °C to room temperature led to nickel-gold complex **50a** and **50b**, which afforded one-component metal complexes **51a** and **51b** after electrocrystallization (Scheme 15) [59].

The obtained one-component molecular conductors with diselenadithiafulvalene skeletons **51a**,**b** showed high three-dimensional conductivity at room temperature [60,61].

**Scheme 16:**
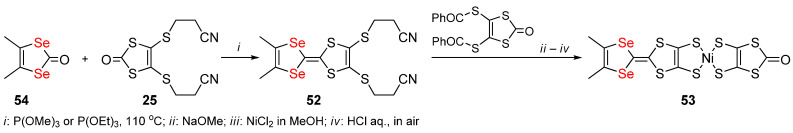
The synthesis of nickel unsymmetrical complex **53** [62].

The compound dimethyldiselenadithiafulvalene **52**, the analog of diselenadithiafulvalene **49**, was obtained by a similar way outlined in Scheme 16. The resulting nickel unsymmetrical complex **53** exhibited a strong third-order non-linear optical response in the visible and near-infrared regions of the spectrum and was regarded as a possible photoconductor [62].

Bis(1,3-dithiole-2-thione-4,5-dithiolato)nickelate, Ni(dmit)_2_, having the complex **53**-like structure, was studied by Naito and colleagues and showed photochemical properties in the composition with *N*,*N*′-ethylene-2,2′-bipyridinium [63] and methyl viologen salts [64].

**Scheme 17:**
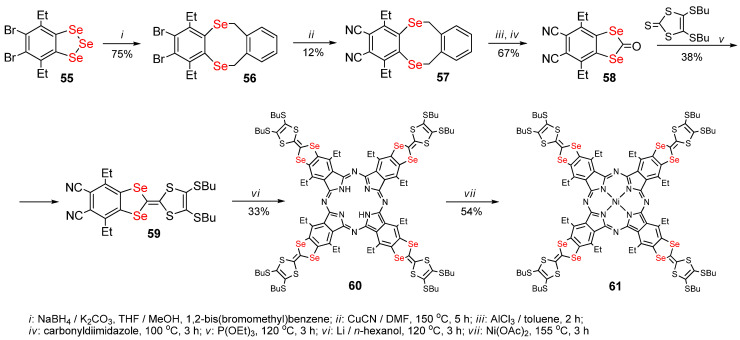
The synthetic approaches to the complexes **60** and **61** [65].

The compound **58** was obtained in 67% yield from 5,6-dibromo-4,7-diethylbenzotriselenole **55** via intermediate compound, 4,5-(o-xylylenediseleno)-3,6-diethyl-1,2-dibromobenzene (**56**) (Scheme 17) [65]. The latter compound, after removing the o-xylylene protecting group, was involved in the reaction with carbonyldiimidazole. The reaction of compound **58** with 4,5-bis(butylthio)-1,3-dithiol-2-thione in triethylphosphite at 120 °C for 3 h led to 3,6-diethylphthalonitrile **59**, containing the dithiadiselenafulvalene moiety. The dithiadiselenafulvalene tetramer-octamethylphthalocyanine **60** was obtained in 33% yield by the treatment of compound **59** with lithium alkoxide at 120 °C. The compound **60** was involved in the reaction with nickel acetate at 155 °C affording a nickel complex **61** (Scheme 17) [65].

Compounds **60** and **61** were used for preparation of electron transfer complexes, which showed a radical cationic character. The charge in the diselenadithiafulvalene structure can be delocalized to the entire molecule [65].

**Scheme 18:**
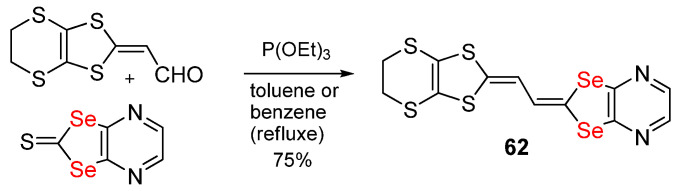
The synthesis of compound **62** [66].

A simple method for the synthesis of tetrathiafulvalene vinylogs, substituted diselenafulvenes, has been developed. Triethylphosphite was added to a solution of the aldehyde and pyrazine-substituted 1,3-diselenole-2-thione in toluene (or benzene) and the mixture was refluxed for 2 h. The target product **62** was isolated in 75% yield (Scheme 18) [66]. This method is applicable to the synthesis of 1,3-diselenoles containing exotic substituents (such as the pyrazine ring), which are sensitive to some highly reactive reagents used in the classical Wittig reaction.

**Scheme 19:**
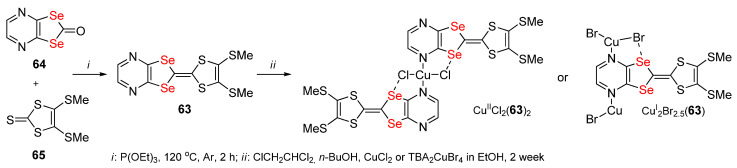
The synthesis of diselenadithiafulvalene **63**, condensed with the pyrazine cycle, and its copper complexes [67].

Diselenadithiafulvalene **63**, condensed with the pyrazine cycle, was synthesized from pyrazino-1,3-diselenole-2-one **64** and 4,5-bis(methylthio)-1,3-dithiol-2-thione **65** (Scheme 19) [67]. A mixture of compounds **64** and **65** was heated in triethylphosphite at 120 °C in a nitrogen atmosphere for 2 h. The copper complexes CuCl_2_(**63**)_2_ and [Cu_2_Br_2.5_(**63**)] were obtained by the method of vertical diffusion. The first complex exhibited the dielectric properties, whereas the second complex showed the properties of semiconductor (Scheme 19) [67].

**Scheme 20:**
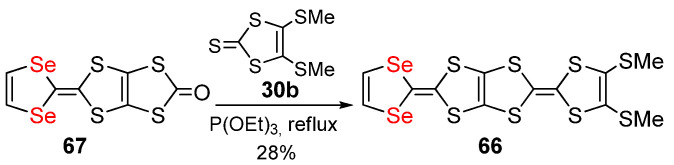
The synthesis of compound **66** [68].

The synthesis of 5-(1,3-diselenole-2-ylidene)-1,3,4,6-tetrathiapentaline (**66**) in 28% yield by the cross-coupling reaction between 1,3-dithiol-2-one derivative **67** condensed with diselenadithiafulvalene and 4,5-bis(methylthio)-1,3-dithiol-2-thione (**30b**) was developed by refluxing the reagents in toluene in the presence of trimethylphosphite (Scheme 20) [68]. The radical-cationic salt (**66**)_4_PF_6_ was studied by the voltammetric method and showed metallic properties down to 5 K.

**Scheme 21:**
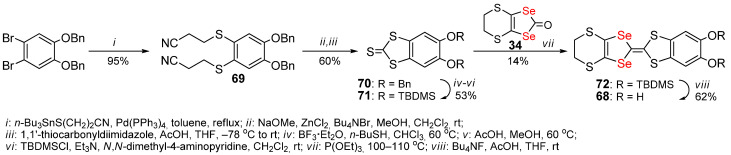
The efficient synthesis of dithiadiselenafulvalene derivatives **68** and **72**, condensed with catechol [69].

The efficient synthesis of catechol-condensed dithiadiselenafulvalene derivative **68** was developed (Scheme 21) [69]. Compound **68** was a new type of molecular *π*-electron donor having two phenolic hydroxyl groups, which was promising for the preparation of charge transfer salts. Treatment of compound **69** with NaOMe and ZnCl_2_ followed by reaction with thiocarbonyldiimidazole under acidic conditions gave 1,3-benzodithiol-2-thione derivative **70** in 60% yield followed by removing benzyl protecting groups by treatment with BF_3_·Et_2_O and BuSH. Subsequent re-protection with *tert*-butyldimethylsilyl group gave compound **71** in 53% yield. Finally, the target compound **68** was obtained in 62% yield by cross-coupling reaction between the product **71** and ketone **34** in triethylphosphite followed by deprotection of the *tert*-butyldimethylsilyl group in compound **72** (Scheme 21) [69].

**Scheme 22:**
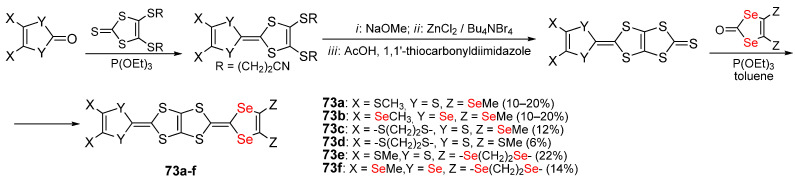
The synthetic route to compounds **73a**–**f [70,71]**.

Selenium-containing tetrathiapentalene condensed compounds with the fulvalene moiety **73a**–**f** were synthesized by three successive cross-coupling reactions (Scheme 22) [70,71]. Iodine salts (**73a**)(I_3_)_5/3_ and (**73b**)(I_3_)_5/3_ showed high conductivity at room temperature [70], whereas salts based on arsenic fluoride (**73c**)(AsF_6_)_0.32_ and (**73f**)(AsF_6_)_0.35_ exhibited semiconductor properties [71].

**Scheme 23:**
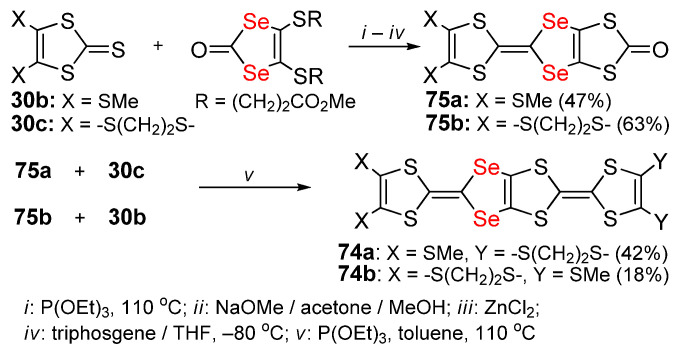
The synthesis of compounds **74a**,**b** [72].

Two new donor molecules of the tetrathiapentalene type, compounds **74a**,**b**, containing two selenium atoms and six sulfur atoms in the heterocyclic skeleton, were synthesized by the cross-coupling reaction depicted in Scheme 23 [72]. This combination of the sulfur and selenium atoms in the heterocyclic scaffold results in a particular type of resistivity: flat resistivity over a wide temperature range for the PF_6_ and AsF_6_ salts of **74a**,**b**, which showed good conductivity [72].

**Scheme 24:**
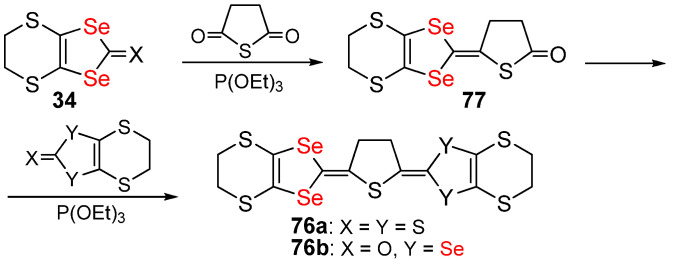
The preparation of the products **76a**,**b** by the cross-coupling reactions [73].

New selenium-containing *π*-extended donors, the products **76a**,**b** were synthesized by the cross-coupling reaction of compound **77** with 4,5-ethylenedithio-1,3-dithiol-2-thione or -2-one **34** (Scheme 24) [73]. The intermediate compound, tetrahydrothiophen-2-one **77**, was obtained by the cross-coupling of 4,5-ethylenedithio-1,3-diselenole-2-one **34** with succinic thioanhydride in the presence of triethylphosphite.

It was found that the PF_6_, AsF_6_, and SbF_6_ salts of the product **76b** exhibited metallic properties down to 2 K, while the PF_6_ and AsF_6_ salts of compound **76a** showed semiconducting behavior.

### 2.3. Halogenated 1,4-Diselenafulvenes and 1,4,5,8-Dithiadiselenafulvalenes

Halogenated sulfur and selenium-containing fulvalenes have attracted much attention in respect to the unique crystal and electronic structures of their cation radical salts [74,75,76]. In contrast to the other halogenated fulvalenes, iodinated diselenafulvalenes have special ability to construct an intermolecular “iodine bond” by interaction of the iodine atom with other functional groups. The physical properties of materials such as organic conductors depend considerably on the crystal structure properties, and introduction of an “iodine bond” is one of the most effective methods of design and crystal engineering in organic conductors [74,75,76].

**Scheme 25:**
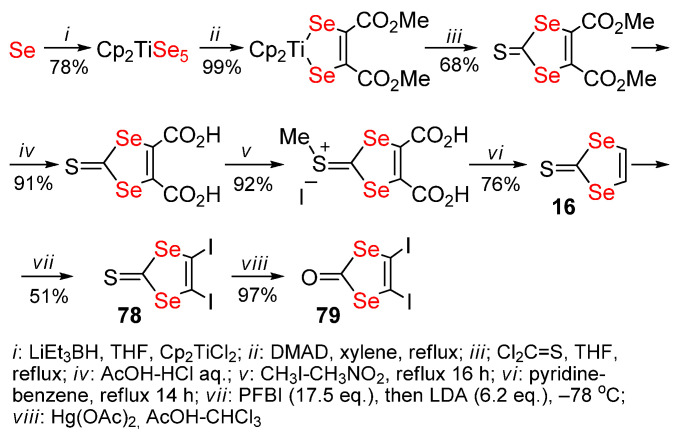
The synthetic route to the products **78** and **79 [74]**.

A new efficient multistep method for the synthetic preparation of 1,3-diselenole-2-thione **16** in 76% yield without the use of toxic carbon diselenide (CSe_2_) was developed (Scheme 25) [74]. Dicyclopentadienyl dichlorotitanium and readily available elemental selenium were used as starting materials in this synthesis. The resulting diselenafulvene **16** was converted into iodo derivatives **78** and **79**. In order to introduce efficiently the iodine atoms and to obtain compounds **78** and **79** in good yields (51% and 97% yields, respectively), a 17-fold excess of perfluorobutyl iodide (PFBI) and a 6-fold excess of lithium diisopropylamide were used (Scheme 25) [74]. The products **78** and **79**, in turn, were regarded as valuable starting compounds for the synthesis of various tetraselenafulvalene derivatives.

**Scheme 26:**
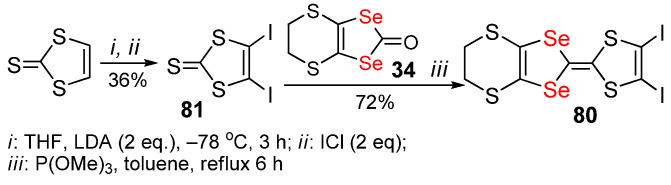
The synthesis of the product **80** [75].

The iodine-substituted analog of dithiadiselenafulvalene, compound **80**, was synthesized in 72% yield by successive iodination of the starting 1,2-dithiol-2-thione with iodine monochloride and the cross-coupling reaction of product **81** with ketone **34** (Scheme 26) [75]. The salt (**80**)_4_[Fe(CN)_5_NO] was prepared by electrocrystallization from a solution of the corresponding donor dithiadiselenafulvalene **80** in dichloromethane and bis(tetraphenylphosphonium)nitroprusside, (PPh_4_)_2_·[Fe(CN)_5_NO], which was used as an electrolyte. It is worth noting that the salt (**80**)_4_[Fe(CN)_5_NO] exhibited semiconducting properties [75].

**Scheme 27:**
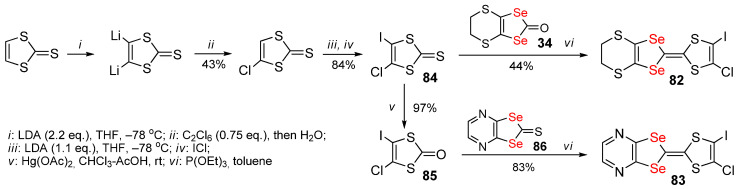
The synthetic approaches to the products **82** and **83 [76]**.

New halogenated diselenadithiafulvalenes **82** and **83**, containing both chlorine and iodine atoms, were synthesized in 44% and 83% yields, respectively, by the reaction sequence shown in Scheme 27 [76]. Intermediate compound **84** was obtained by successive lithiation by lithium diisopropylamide, chlorination with hexachloroethane, and iodination of the lithium derivative of 1,2-dithiol-2-thione with iodine monochloride. Compound **84** was converted to ketone **85** using the Hg(OAc)_2_-CHCl_3_-AcOH system. Intermediate compounds **84** and **85** were further involved in the cross-coupling reactions with the corresponding ketone **34** and thione **86**. Studies of the properties of compounds **82** and **83** showed that the chlorine atom mainly contributes to the electronic properties within one molecule, and the iodine atom to intermolecular interaction through the iodine bond. Appropriate application of the different roles of halogen atoms can be useful for the development of new supermolecular organic conductors based on these compounds (Scheme 27) [76].

**Scheme 28:**
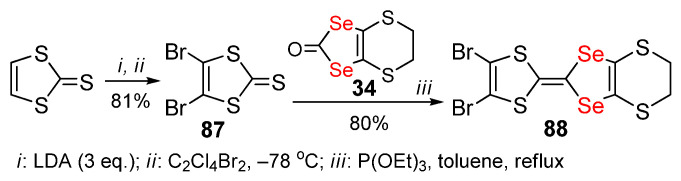
The synthesis of compound **88** **[77]**.

The cross-coupling reaction of 4,5-dibromo-1,3-dithiol-2-thione **87** with 4,5-ethylenedithio-1,3-diselenole-2-one **34** afforded 4,5-dibromo-4’,5’-ethylenedithiodiselenadithiafulvalene **88** in 80% yield (Scheme 28) [77]. Compound **88** exhibited electron-donating properties. A charge-transfer complex of compound **88** with tetracyanoquinodimethane, (**88**)_2_[TCNQ], was obtained by slow evaporation of dichloromethane from a solution of these compounds [77].

The authors noted that bromo derivatives of selenafulvalenes were more available compounds compared to the analogous iodo derivatives. However, the bromo derivatives also display donor abilities and can be used for the preparation of charge-transfer complexes [77].

### 2.4. Non-Condensed 1,4,5,8-Tetraselenafulvalenes

**Scheme 29:**
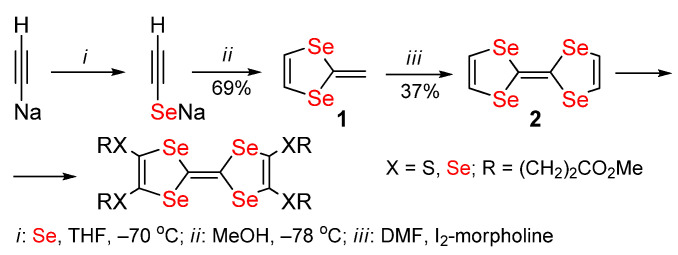
The synthesis of diselenafulvene **1** and tetraselenafulvalene **2** based on selenium and sodium acetylide [78].

Convenient and practical synthetic procedures for the preparation of diselenafulvene **1** and tetraselenafulvalene **2** based on selenium and sodium acetylide were developed (Scheme 29) [78]. This approach has the advantage of using cheap, non-toxic selenium powder as the selenium source and commercially available sodium acetylide in xylene light mineral oil. 

The authors emphasized that only two steps were required for the synthesis of the target compound by this method, which was suitable for laboratory-scale preparation, about 7 g of diselenafulvene **1** and more than 2 g of fulvalene **2** can be obtained by these two experiments under laboratory conditions [78]. The authors also noted that in combination with previously developed methods (the functionalization of tetraselenafulvalene **2** as protected thiolate or selenolate moieties followed by their deprotection/realkylation chemistry), the present approach paved a practical way to various heterocycle-fused tetraselenafulvalene type donors, which can be used to produce superconducting radical cation salts (Scheme 29) [78].

Electrochemical oxidation of tetraselenafulvalene **2** in the presence of potassium nitroprusside K_2_[FeNO(CN)_5_]∙2H_2_O and 18-crown-6 using nitrobenzene as a solvent gave a new single-crystal radical cationic salt (**2**)_7_[FeNO(CN)_5_]_2_, which showed the properties of a conductor at room temperature and 130 K [79].

**Scheme 30:**
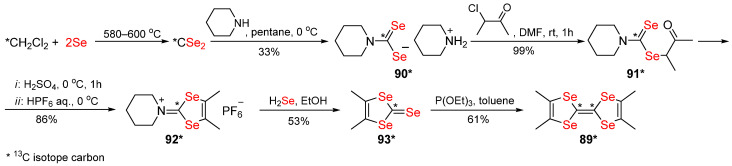
The synthetic route to 4,4’,5,5’-tetramethyl Δ^2,2^-bis-1,3-diselenole 89*, doubly labeled with ^13^C isotope [80].

Efficient syntheses of tetramethyltetraselenafulvalene (**89**) were developed in the last century [4,5,6]. An interesting method for the preparation of tetramethyltetraselenafulvalene, doubly labeled with ^13^C isotope at the positions 2 and 2’ (4,4’,5,5’-tetramethyl Δ^2,2^-bis-1,3-diselenole 89*) was described (Scheme 30) [80]. Labeled with ^13^C isotope carbon diselenide was obtained from ^13^C-dichloromethane at 580–600 °C. The ^13^C-carbon diselenide, after cooling to room temperature and dissolving in pentane, was reacted with piperidine at 0 °C leading to piperidinium 1-piperidine ^13^C-diselenocarbamate 90* in 33% yield. 

The reaction of compound 90* with 3-chloro-2-butanone in DMF for 1 h at room temperature gave 1-piperidine ^13^C-carbodiselenoic acid, 1-methyl-2-oxopropyl ester 91* in quantitative yield (Scheme 30) [80]. Diselenocarbamate 91* was successively treated with concentrated H_2_SO_4_ and 60% aqueous HPF_6_ at 0 °C to form 2-(1-piperidinium)-2-(^13^C)-4,5-dimethyl-1,3-diselenole hexafluorophosphate 92* in 86% yield. Labeled with ^13^C isotope 4,5-dimethyl-1,3-diselenole-2-selone 93* was prepared in 53% yield by the reaction of compound 92* with hydrogen selenide in ethanol. The target product 89* in 61% yield was obtained by the classical method: the cross-coupling reaction in the presence of triethylphosphite (Scheme 30) [80].

The single-site ^13^C-enriched tetramethyltetraselenafulvalene was obtained starting from 4,5-dimethyl-1,3-diselenole-2-one [81]. Correlation between non-Fermi-liquid behavior and antiferromagnetic fluctuations in superconducting (TMTSF)_2_PF_6_ salt was studied using ^13^C-NMR spectroscopy [81].

**Scheme 31:**
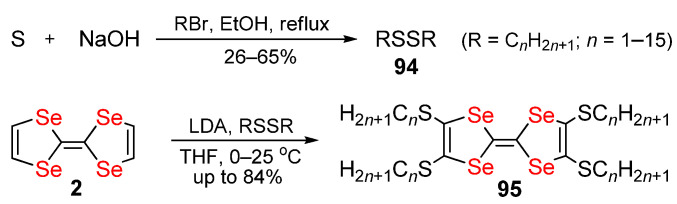
The synthesis of tetrakis(alkylthio)tetraselenafulvalene compounds **95** (*n* = 1–15) from TSF and dialkyl disulfides **94** [82].

A series of tetrakis(alkylthio)tetraselenafulvalene compounds **95** (*n* = 1–15) was synthesized in up to 84% yield by a one-step reaction of dialkyl disulfides **94** with tetralithiated tetraselenafulvalene **2** (Scheme 31) [82]. Compounds **95** were found to be weak electron-donating molecules and to show low dark conductivity. At the same time, as the number of methylene groups increased, the electrical conductivity increased due to the presence of high-dimensional conduction paths. The resulting compounds were highly soluble in organic solvents [82].

The authors noted that the compounds **95** were good candidates for the field-effect transistor channel based on the advantageous features: low dark conductivity, low donor ability, on-site Coulomb repulsion energy, high-dimensional *π*-electron structure, and high solubility in organic solvents [82].

**Scheme 32:**
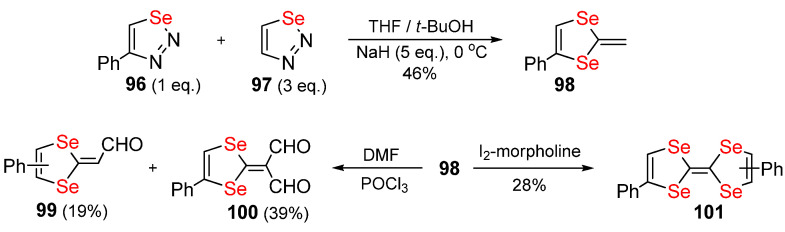
The synthesis of compounds **99**–**101** [83].

The use of one equivalent of phenylselenadiazole **96** and three equivalents of selenadiazole **97** in the reaction of these reagents in a mixture of THF and *tert*-butanol at 0 °C in the presence of five equivalents of sodium hydride led to diselenafulvene **98** in 46% yield. The latter compound was successfully formylated with the formation of compounds **99** and **100** by the Vilsmeier–Haack reaction (Scheme 32) [83].

The products **99** and **100**, bearing the aldehyde group, can serve as precursors of the vinylogs of tetraselenafulvalene derivatives. The iodine-morpholine reagent was used to convert fulvene **98** to diphenyltetraselenafulvalene **101** (a mixture of *E*- and *Z*-isomers) in 28% yield (Scheme 32) [83].

### 2.5. Condensed 1,4,5,8-Tetraselenafulvalenes

**Scheme 33:**
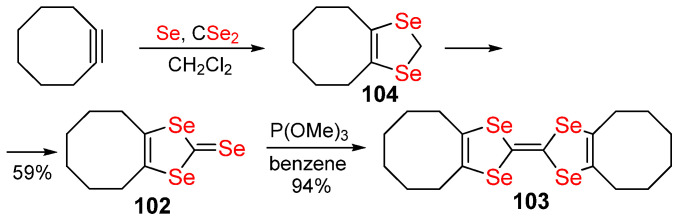
The synthesis of tetraselenafulvalene **103 [84]**.

The reaction of cyclooctyne with carbon diselenide in the presence of red selenium in boiling dichloromethane afforded cycloocteno[1,2-*d*]1,3-diselenole-2-selone **102** (59% yield), which was converted into tetraselenafulvalene **103** in 94% yield by the treatment with trimethylphosphite in boiling benzene (Scheme 33) [84]. The formation of selone **102** can be rationalized by addition of carbon diselenide to cyclooctyne to form 1,3-diselenole-2-ylidene **104**, which then reacts with elemental selenium or can capture selenium atom from the carbon diselenide molecule yielding compound **102** (Scheme 33) [84]. This reaction successfully competed with the carbene dimerization with the formation of tetraselenafulvalene **103** if the process was carried out in the absence of elemental red selenium.

**Scheme 34:**
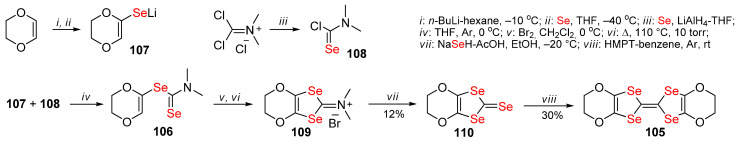
The convenient synthetic approach to bis(ethylenedioxy)tetraselenafulvalene **105** [85].

The convenient synthesis of bis(ethylenedioxy)tetraselenafulvalene **105** without the use of toxic reagents such as carbon diselenide and hydrogen selenide was developed (Scheme 34) [85]. The key intermediate **106** was synthesized by the reaction between lithium selenolate **107** and *N*,*N*-dimethylselenocarbamoyl chloride **108** in THF at 0 °C under argon. Both compounds **107** and **108** can be prepared based on elemental selenium powder. Diselenocarbamate **106** was quantitatively converted to iminium salt **109**, which was used to prepare selone **110** in the NaSeH-AcOH system. The synthesis of the target tetraselenafulvalene **105** (30% yield) was carried out by a coupling reaction under very mild conditions in benzene using hexamethylphosphorous triamid (HMPA) at room temperature under argon (Scheme 34) [85]. The new donor compound **105** exhibited sufficient solubility in common organic solvents and the ability to form CH…O hydrogen bonds. Its electrochemical properties were found to be promising for obtaining new organic metals, including superconductors [85].

**Scheme 35:**
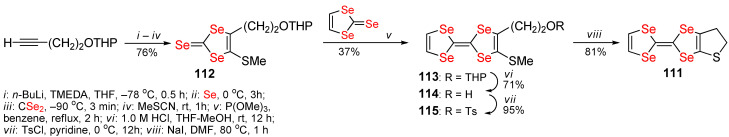
The synthetic route to ethylenethio-1,4,5,8-tetraselenafulvalene **111 [86]**.

A condensed derivative of tetraselenafulvalene, ethylenethio-1,4,5,8-tetraselenafulvalene **111**, was synthesized as a new promising electron donor (Scheme 35) [86]. The key intermediate was the 1,3-diselenole-2-selone derivative **112**, easily prepared from commercially available tetrahydropyranyl-protected 3-butyn-1-ol. Using a conventional cross-coupling reaction with 1,3-diselenole-2-selone, the desired product **113** was obtained in 37% yield. Then, the tetrahydropyranyl-protecting group of compound **113** was removed by treatment with dilute hydrochloric acid, and the resulting alcohol **114** (71% yield) was converted into tosylate **115** in 95% yield.

The formation of the dihydrothiophene ring was achieved by a transalkylation reaction at the sulfur atom in the presence of sodium iodide in DMF, which resulted in the desired ethylenethiotetraselenafulvalene **111** in 81% yield (Scheme 35) [86]. The use of electrocrystallization method for compound **111** gave highly conductive radical cationic salts with a number of counter anions such as I_3_^−^, Cl^−^, Br^−^, and AuI_2_^−^ in a 2:1 donor-acceptor ratio.

**Scheme 36:**
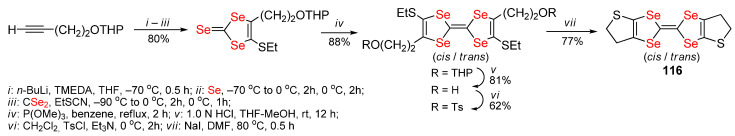
The synthetic approach to bis(ethylenethio)-1,4,5,8-tetraselenafulvalene **116 [87]**.

In a similar manner, bis(ethylenethio)-1,4,5,8-tetraselenafulvalene **116** was prepared (77% yield), which formed a highly conductive molecular complex with tetracyanoquinodimethane (TCNQ) in a 1:1 donor-acceptor ratio (Scheme 36) [87]. These materials retained metallic properties down to 60 K.

**Scheme 37:**
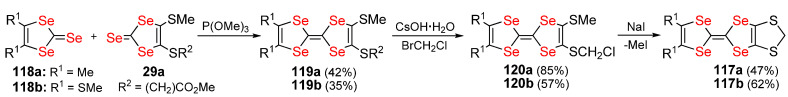
The preparation of derivatives **117a**,**b** [52].

A three-stage synthesis of new donors, dimethyl and bis(methylthio) derivatives of methylenedithiotetraselenafulvalene **117a**,**b**, was developed (Scheme 37) [52]. Cross-coupling reactions between 1,3-diselenole-2-selones **118a**,**b** and 4-methylthio-5-(2-methoxycarbonylethylthio)-1,3-diselenole-2-selone **29a** in the presence of trimethylphosphite gave intermediate products **119a**,**b** in 42% and 35% yields, respectively. Removing the protective 2-methoxycarbonylethylthio group in compounds **119a**,**b** with cesium hydroxide in DMF solution and in situ treatment with bromochloromethane led to the corresponding 2-methylthio-3-chloromethylthio derivatives of tetraselenafulvalene **120a**,**b** in 85% and 57% yields, respectively. The transalkylation at the sulfur with the ring-closure reaction were carried out by treatment with sodium iodide to give derivatives **117a**,**b** in 47% and 62% yields, respectively (Scheme 37) [52].

The obtained compounds showed good electron-donating properties. Based on compound **117a**, three radical cation salts were obtained. Salts with I_2_Br^−^ and AsF_6_^−^ anions exhibited semiconducting properties, while the PF_6_^−^ salt displayed metallic conductivity down to 130 K [52].

**Scheme 38:**
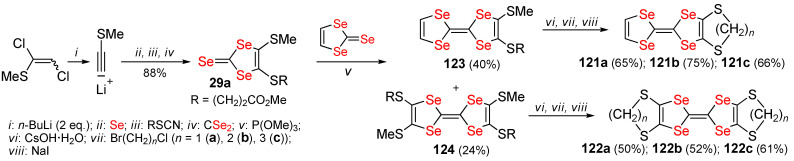
The synthesis of tetraselenafulvalenes derivatives **121a**–**c** and **122a**–**c** [88].

A general synthetic approach to a series of alkylenedithio- (**121a**–**c**, 65%, 75%, and 66% yields, respectively) and bis(alkylenedithio)tetraselenafulvalenes **122a**–**c** (50%, 52%, and 61% yields, respectively) was developed (Scheme 38) [88]. Key intermediate compounds **123** and **124** were readily prepared by phosphite-activated reactions of 4-methylthio-5-(2-methoxycarbonylethylthio)-1,3-selenole-2-selone **29a**. The latter compound was obtained in 88% yield from a fairly stable, readily available, and cheap reagent, 1,2-dichloro-1-methylthioethane, by treating it with butyl lithium in situ to form lithium 2-methylthioacetylide, followed by the reaction of the acetylide with selenium, methyl-3-thiocyanatopropionate and carbon diselenide (Scheme 38) [88].

The obtained compounds were found to be excellent electron donors for the preparation of organic conductors [88].

**Scheme 39:**
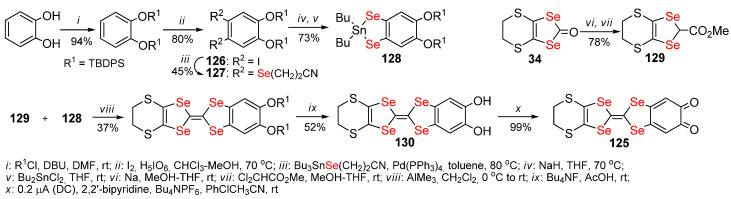
The synthetic route to catechol-condensed tetraselenafulvalene **130** [89].

Benzoquinone-fused ethylenedithiotetraselenafulvalene **125** was synthesized by the reaction sequence depicted in Scheme 39 [89]. The key intermediate compound **128** was obtained in 73% yield by a four-step synthesis from catechol: protection of two hydroxyl groups with *tert-*butyldiphenylsilyl groups, diiodination, the Stille cross-coupling reaction with Bu_3_SnSe(CH_2_)_2_CN followed by treatment with NaH and Bu_2_SnCl_2_. In the presence of AlMe_3_, the reaction of compound **128** with methyl ester **129** obtained from ketone **34** and subsequent deprotection of hydroxyl groups gave catechol-condensed tetraselenafulvalene **130** in 52% yield. Electrochemical oxidation of compound **130** in the presence of 2,2’-bipyridine afforded ortho-benzoquinone-fused ethylenedithiotetraselenafulvalene **125** in quantitative yield (Scheme 39) [89].

**Scheme 40:**
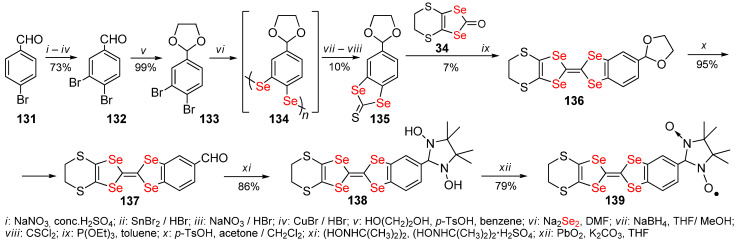
The synthesis of new organic spin-polarized donor **139**, which exhibited ferromagnetic properties [90].

Nitration of para-bromobenzaldehyde **131** with sodium nitrate in concentrated sulfuric acid giving 4-bromo-3-nitrobenzaldehyde and reduction of the nitro group by subsequent Sandmeyer reaction made it possible to obtain 3,4-dibromobenzaldehyde **132** in 73% yield (Scheme 40) [90]. The aldehyde **132** was quantitatively converted to 2-(3,4-dibromophenyl)-1,3-dioxolane **133** to protect the formyl group in further reactions. Poly(diselenide) **134** was prepared by the reaction between dibromo compound **133** and sodium diselenide in DMF. The reduction of polymer **134** with sodium tetrahydroborate followed by treatment with thiophosgene made it possible to obtain the key 5-(1,3-dioxolan-2-yl)-benzo-1,3-diselenole-2-thione **135**. The cross-coupling reaction between thione **135** and 4,5-(ethylenedithio)-1,3-diselenole-2-one **34** in triethylphosphite led to tetraselenafulvaleneacetal derivative **136**, which was hydrolyzed to produce formyl derivative **137**. The latter compound was involved in the reaction with 2,3-dimethyl-2,3-bis(hydroxyamino)butane in the presence of its sulfuric acid salt as a catalyst giving cyclic bis(hydroxylamine) **138** in 86% yield as a radical precursor. Compound **138** was converted by oxidation with lead dioxide into a new organic spin-polarized donor **139** in 79% yield, which exhibited ferromagnetic properties (Scheme 40) [90].

New tetraselenafulvalenes derivatives find application in the synthesis of organic spin-polarized donors [90].

**Scheme 41:**
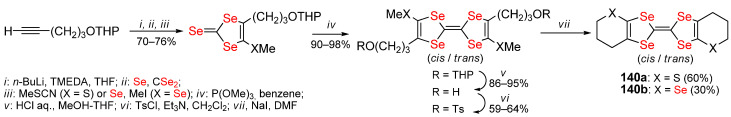
The efficient approach to new fused heterocycles **140a** and **140b [91,92]**.

The efficient approach to new fused heterocycles, bis(propylenethio)tetraselenafulvalene **140a** (60% yield) and bis(propyleneseleno)tetraselenafulvalene **140b** (30% yield) based on tetrahydropyran-protected pent-4-yn-1-ol as the starting compound is outlined in Scheme 41 [91,92].

Various types of highly conductive radical-cationic salts were prepared based on tetraselenafulvalenes **140a**,**b**. For example, the PF_6_, AsF_6_, and FeCl_4_ salts retained metallic properties down to the liquid helium temperature [91,92].

**Scheme 42:**
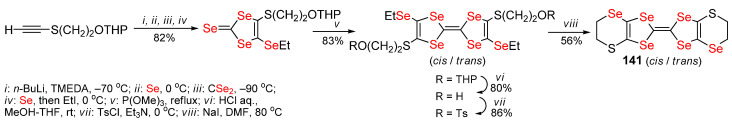
Synthesis of tetraselenafulvalene derivative **141** from tetrahydropyranyl-protected acetylene [92,93].

Tetraselenafulvalene derivative **141** was obtained in 56% yield using tetrahydropyranyl-protected acetylene containing a thioethyl fragment as the starting compound by the approach illustrated in Scheme 42 [92,93].

**Scheme 43:**
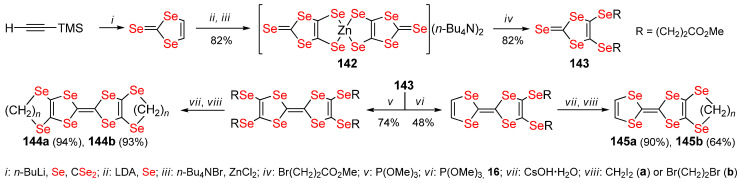
The efficient and practical synthetic method for the preparation of condensed electron donors of the tetraselenafulvalene type **144a**,**b** and **145a**,**b [94]**.

The efficient and practical synthetic method for the preparation of condensed electron donors of the tetraselenafulvalene type **144a**,**b** and **145a**,**b** in up to 94% yield was developed by the approach depicted in Scheme 43 using tetrabutylammonium 4,5-bis(2-selenoxo-1,3-diselenole-4,5-diselenolate)zincate **142** (82% yield) as the intermediate compound [94].

The obtained compounds **144a**,**b** and **145a**,**b** served as efficient electron donors for preparation of organic conducting materials [94].

**Scheme 44:**
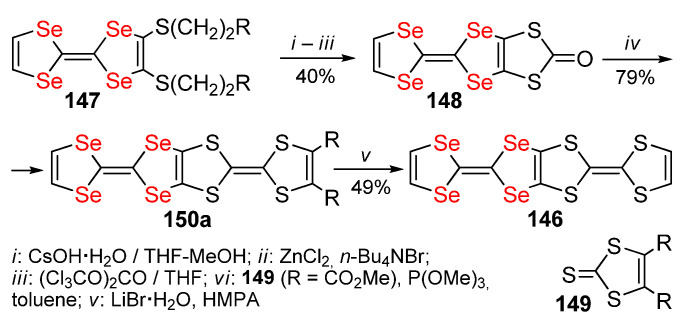
Synthesis of compound **146**, containing tetrathiafulvalene structure condensed with the tetraselenafulvalene scaffold [95].

A new very interesting donor compound, 2-(1,3-diselenole-2-ylidene)-5-(1,3-dithiol-2-ylidene)-1,3-diselena-4,6-dithiapentalene **146**, containing tetrathiafulvalene structure condensed with the tetraselenafulvalene scaffold, was synthesized in 49% yield (Scheme 44) [95]. The TSF derivative **147** was sequentially treated with CsOH·H_2_O, with ZnCl_2_, *n*-Bu_4_NBr, and then with triphosgene to obtain compound **148** (40% yield). The cross-coupling reaction between **148** and 4,5-bis(methoxycarbonyl)-1,3-dithiol-2-thione (**149**, two equivalents) in the presence of trimethyl phosphite at reflux in toluene gave bis(methoxycarbonyl) derivative **150a** in 79% yield. The product **146** was obtained in 49% yield by demethoxycarbonylation of compound **150a** with excess LiBr·H_2_O in hexamethylphosphorous triamide (Scheme 44) [95]. The radical cationic salt (**146**)ReO_4_ exhibited low conductivity with a high activation energy.

**Scheme 45:**
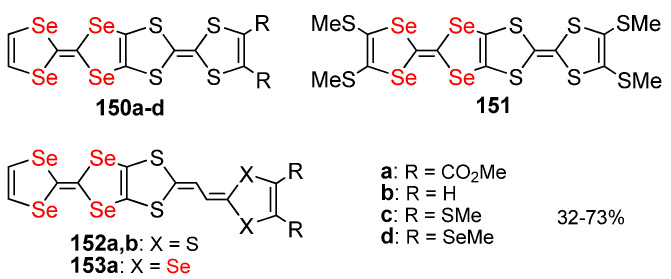
The synthesis of donor compounds **150a**–**d**, **151**, **152a,b** and **153a** [96].

A number of derivatives of bis-condensed donors **150a**–**d** and **151**, as well as their vinyl analogs **152a**,**b** and **153a**, were synthesized in 32–73% yields using corresponding intermediate zincates (Scheme 45) [96].

The donor compounds **150a**, **152a**, and **153a** form highly conductive complexes with tetracyanoquinodimethane (TCNQ) and salts with I_3_ with very low activation energies of 0.0094–0.040 eV (Scheme 45) [96].

### 2.6. Other 1,4-Selenafulvene and 1,4,5,8-Selenafulvalene Derivatives

**Scheme 46:**
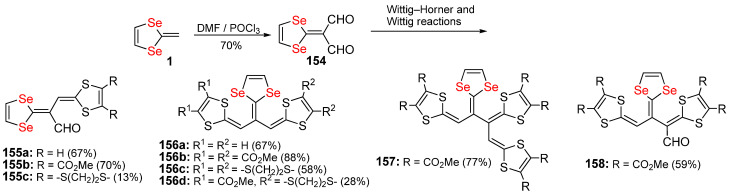
Synthesis of bicyclic and polycyclic compounds **155a**–**c**, **156a**–**d**, **157**, and **158** including vinylgues of the dendralene type [97].

The intermediate 2-(1,1-diformylmethylene)-1,3-diselenole **154** was obtained in 70% yield from 2-methylene-1,3-diselenole **1** by the Vilsmeyer–Haack reaction (Scheme 46) [97]. The dialdehyde **154** was further condensed with dithiolium phosphonium bromides and dithiolium phosphonates in the presence of a base. As a result, a number of polycyclic selenium-containing tetrathiafulvalene vinylogues of the dendralene type **155a**–**c**, **156a**–**d**, **157**, and **158** bearing a 1,3-diselenole moiety were obtained in good yields by the Wittig and Wittig–Horner reactions (Scheme 46) [97]. The obtained products showed electrochemical activity.

**Scheme 47:**
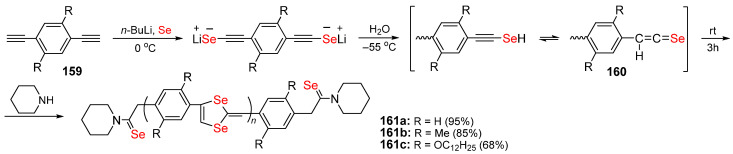
Synthesis of electron-donating *π*-conjugated polymers **161a**–**c** [98].

The selenoketenes **160** were obtained by deprotonation of aromatic diynes **159** with *n*-BuLi, subsequent addition of elemental selenium at 0 °C, and reaction with water in the temperature range from –55 °C to room temperature for 3 h (Scheme 47) [98]. The resulting intermediates **160** in situ were subjected to cycloaddition polymerization to produce electron-donating *π*-conjugated polymers **161a**–**c** with a 1,4-diselenafulvene moiety in 95%, 85%, and 68% yields, respectively.

The solubility of the polymers depends on their structure. The attachment of long alkyl chains enhances solubility in non-polar solvents. The resulting polymers exhibited electron-donating properties, as did their soluble charge-transfer complexes with tetracyanoquinodimethane [98].

**Scheme 48:**
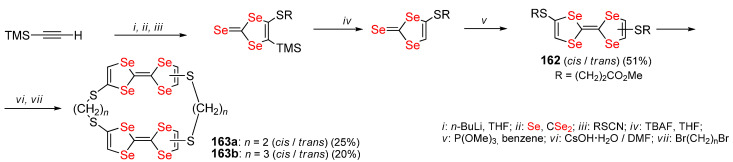
The synthetic approach to two-bridged tetraselenafulvalenophanes **163a**,**b** from trimethylsilylacetylene [99].

Two-bridged tetraselenafulvalenophanes **163a**,**b** were efficiently synthesized from trimethylsilylacetylene by the synthetic approach presented in Scheme 48 [99]. Using sequential deprotection and realkylation of the bis-thiolate tetraselenafulvalene building block **162** (51% yield), two-bridged tetraselenafulvalenophanes **163a**,**b** were efficiently synthesized in 25% and 20% yields, respectively (Scheme 48) [99]. The radical cationic salt **163b** with the Au(CN)_2_^−^ anion exhibited very high conductivity at room temperature.

**Scheme 49:**
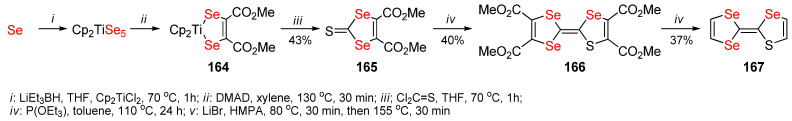
The synthetic route to triselenathiafulvalene **167** [100].

The synthesis of 4,5-dicarbomemethoxy-1,3-diselenole-2-thione **165** in 43% yield was developed from the intermediate titanocene pentaselenide **164** and elemental selenium, avoiding the use of highly toxic carbon diselenide (Scheme 49) [100].

The cross-coupling of two half-blocks of compound **165** in the presence of triethylphosphite led to the unsymmetrical derivative tetracarbomethoxytriselenathiafulvalene derivative **166** in 40% yield. As a result of the sulfur-selenium “scrambling” in the presence of the electron-withdrawing group in the positions 4 and 5 of the diselenole ring, a mixed thiaselenafulvene core was generated. Unsubstituted triselenathiafulvalene **167** was obtained in 37% yield by decarbomethoxylation of compound **166** (Scheme 49) [100].

The charge transfer complex of triselenathiafulvalene **167** with tetracyanoquinodimethane showed a high degree of anisotropic conductivity in the polycrystalline sample, which decreased with increasing temperature [100].

## 3. The Synthesis of 1,4-Ditellurafulvene and 1,4,5,8-Tetratellurafulvalene Derivatives

The efficient synthetic approaches to 1,4-ditellurafulvenes and 1,4,5,8-tetratellurafulvalenes are less developed than to the corresponding selenium derivatives and a number of the obtained derivatives of these two classes of organotellurium compounds is significantly smaller than that of known 1,4-diselenafulvenes and 1,4,5,8-tetraselenafulvalenes.

**Scheme 50:**
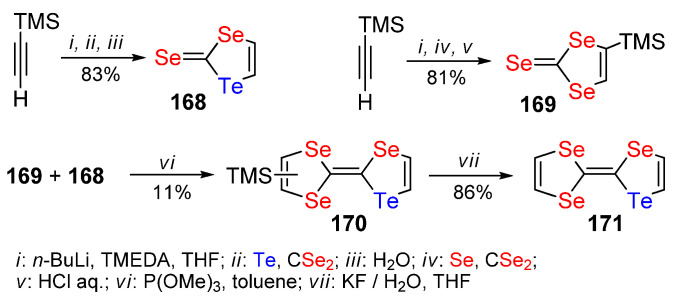
The convenient synthesis of triselenatellurafulvalenes **170** and **171** based on trimethylsilylacetylene [101].

The cross-coupling reaction of 1,3-selenatellurole-2-selone **168** and trimethylsilyl derivative of 1,3-diselenole-2-selone **169** gave triselenatellurafulvalene with trimethylsilyl group **170**, which was separated by gel permeation chromatography in 11% yield (Scheme 50) [101]. The trimethylsilyl group was then easily removed by the desilylation reaction with potassium fluoride in an aqueous THF solution giving triselenatellurafulvalene **171** in 86% yield. The complex of the product **171** with tetracyanoquinodimethane showed high conductivity at room temperature and retained metallic properties at 85 K [101].

**Scheme 51:**
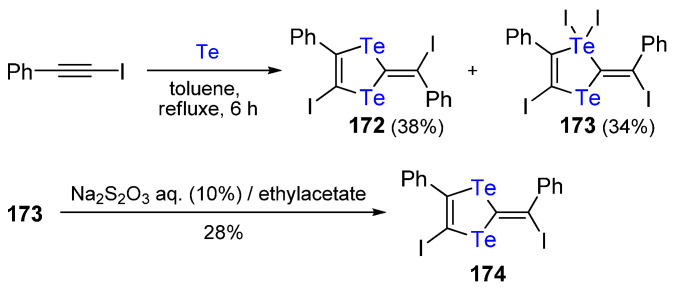
The synthesis of 1,4-ditellurafulvene derivative **172**, **173**, and **174** from phenyliodoacetylene and tellurium [102].

The thermal reaction between phenyliodoacetylene and powdered tellurium gave a mixture of (*E*)-1,4-ditellurafulvene derivative **172** and its iodinated isomer (*Z*)-1,1-diiodo-1,4-ditellurafulvene **173** by refluxing the reaction mixture in toluene for 6 h. After the chromatographic separation of these two isomers, (*Z*)-1,1-diiodo-1,4-ditellurafulvene **173** was reduced by aqueous solution of sodium thiosulfate to corresponding (*Z*)-ditellurafulvalene **173** with the retention of stereoconfiguration (Scheme 51) [102].

**Scheme 52:**
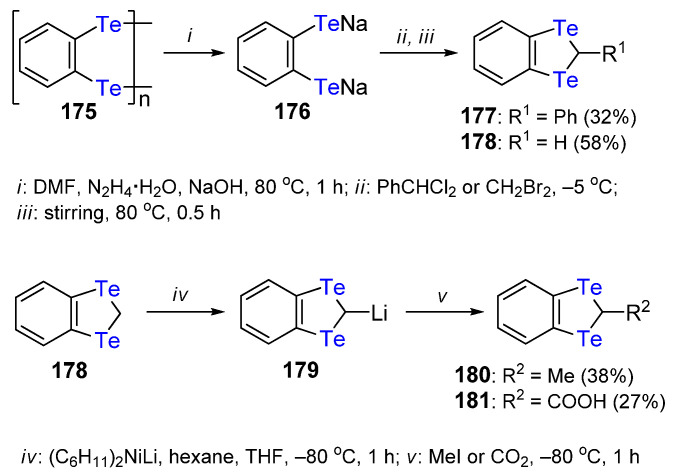
The synthesis of benzo-1,3-ditelluroles **177** and **178**, 2-methylbenzo-1,3-ditellurole (**180**) and benzo-l,3-ditellurole-2-carboxylic acid **181** [103].

Reduction of poly(*o*-phenylene ditelluride) **175** with the hydrazine hydrate/sodium hydroxide system in DMF led to generated disodium o-benzeneditellurolate **176**, which reacted with benzylidene chloride or dibromomethane to give 2-phenylbenzo-1,3-ditellurole **177** or benzo-1,3-ditellurole **178** in 32% and 58% yields, respectively (Scheme 52) [103].

In contrast to benzo-1,3-dithiole and its 2-substituted derivatives, which can be lithiated with butyllithium, the C-Te bond in benzo-1,3-ditellurole **178**, as well as in its noncyclic analogs, diorganyl tellurides, R_2_Te, is cleaved by butyllithium. As a result of the reaction of heterocycle **178** with *n*-BuLi, even at low temperatures, a complex mixture of various diorganyl tellurides is formed [103].

Although 2-lithiobenzo-1,3-ditellurole **179** was generated in rather low yield by treating a solution of heterocycle **178** with lithium dicyclohexylamide in THF, 2-methylbenzo-1,3-ditellurole **180** was obtained in 38% yield by the reaction of 2-lithiobenzo-1,3-ditellurole **179** with methyl iodide. The carbonylation of compound **179** with carbon dioxide gave benzo-l,3-ditellurole-2-carboxylic acid **181** in 27% yield (Scheme 52) [103].

**Scheme 53:**
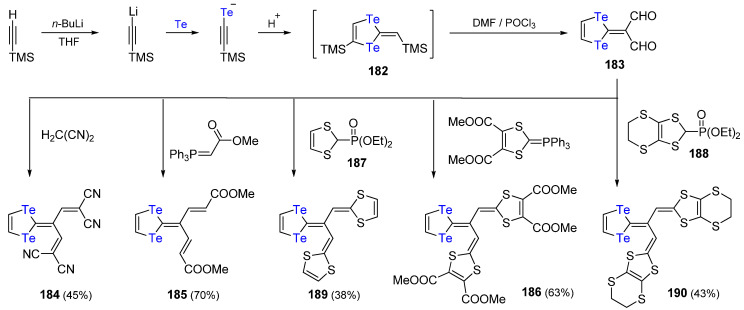
Efficient synthetic routes to compounds **184** and **185** and dendralene **186**, **189**, and **190** by condensation reactions of dialdehyde **183 [104]**.

The successful synthesis of dendralenic ditellurole-containing compounds including dendralenes was developed based on trimethylsilylacetylene and elemental tellurium (Scheme 53) [104]. Trimethylsilylacetylene was lithiated with butyllithium and lithium trimethylsilylacetylide reacted with tellurium to give tellurolate, which was protonated leading to unstable intermediate **182**. Crude compound **182** was subjected to the Vilsmeier–Haack reaction to afford stable desired dialdehyde **183**. The latter compound was condensed with malononitrile and carbomethoxymethyl phosphorane to give products **184** and **185** in 45% and 70% yields, respectively. Dialdehyde **183** was efficiently reacted with 4,5-dicarbomethoxy-1,3-dithiole phosphorane in the presence of sodium hydride to produce dendralene **186** in 63% yield. In like fashion, phosphonates **187** and **188** afforded dendralenes **189** and **190** in 38% and 43% yields, respectively, upon condensation with dialdehyde **183** in the presence of a base (Scheme 53) [104].

**Scheme 54:**
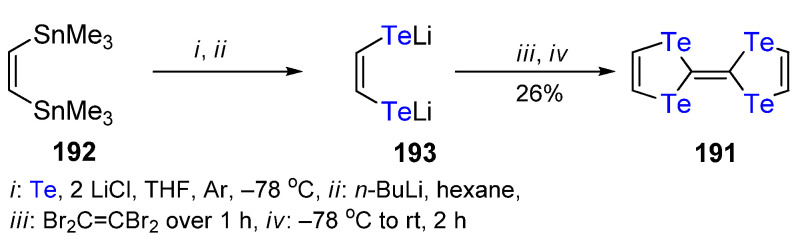
The convenient method for the preparation of tetratellurafulvalene **191** [105].

A systematic study of the synthesis of the *π*-donor tetratellurafulvalene **191** made it possible to increase the yield of the purified fulvalene product from about 12% to quite reproducible values reaching 26% (if tetrabromoethene is used at the final stage of cyclization). The optimized procedure for the synthesis of tetratellurafulvalene **191** is as follows (Scheme 54) [105]. A solution of *n*-BuLi in hexane was added to a suspension of distannan **192**, tellurium, and LiCl in THF cooled to −78 °C over 30 min in an argon atmosphere. After additional stirring for 45 min, tetrabromoethene in THF was added to this suspension containing ditellurolate **193** over 1 h followed by stirring at −78 °C for 2 h. The crude product, after isolation from the reaction mixture, was purified by column chromatography on silica gel under argon giving tetratellurafulvalene **191** in 26% yield (Scheme 54) [105].

This method of synthesis makes it possible to reliably obtain useful amounts of compound **191** and allowed to contribute to the development of studying the radical cation complexes and charge-transfer salts based on tetratellurafulvalene and to investigate its potential as a building block for the preparation of functionalized electron donor compounds [105].

## 4. Application of 1,4,5,8-Tetraselenafulvalene Derivatives and Their Tellurium Analogs for Materials Sciences

As shown by the literature data, 1,4-diselenafulvene derivatives serve as main building blocks for construction of 1,4,5,8-tetraselenafulvalenes, which find augmenting application in the preparation of materials with varying degrees of conductivity and various properties.

**Scheme 55:**
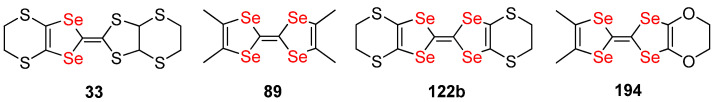
Well-known tetraselenafulvalenes and diselenadithiafulvalene derivatives, which are effective as electron-donor compounds and commonly used for the preparation of conductive materials.

The well-known and frequently used tetraselenafulvalene *π*-donors molecules are bis(ethylenedithio)diselenadithiafulvalene **33**, tetramethyltetraselenafulvalene **89**, bis(ethylenedithio)tetraselenafulvalene **122b**, and dimethyl(ethylenedioxy)tetraselenafulvalene **194** (Scheme 55).

Compound **33** (BEDT-STF) is a very important unsymmetrical donor that was used to obtain the organic charge-transfer complex (**33**)_2_I_3_ exhibiting unique properties to form Dirac electron states under ambient pressure [20,21,22,31,32,53,54,55,56,57].

Recently, much attention has been paid to Dirac electronic systems. However, most of these studies are theoretical (e.g., quantum chemical calculations by DFT methods) due to the limited availability of the relevant materials. The Dirac electron systems (DES) are characterized by massless electrons with relativistic behavior and high speed (1/100–1/1000th of the velocity of light). Previously, similar relativistic behavior of electrons was observed in graphene. In fact, DES were initially found in graphene and some inorganic compounds [22,55]. It should be noted that organic DES exhibit important advantages over their inorganic counterparts. For example, in contrast to inorganic DES, most organic DES are characterized by a clearly defined crystal structure and chemical stoichiometry. However, the majority of organic DES shows the properties of Dirac electron states only under high pressure. In contrast to this observation, the organic charge-transfer complex (**33**)_2_I_3_ exhibits unique properties to form Dirac electron states under ambient pressure [32,53,54,55,56,57]. Based on studies of the electrical, magnetic, optical, and structural properties of (**33**)_2_I_3_ under ambient pressure, it was established that this salt possesses a band structure characterized with Dirac cones, which was in good agreement with the quantum chemical calculations [20,21,22,31,32,53,54,55,56,57]. In fact, this salt is a unique object for research, which can provide an important insight into the properties of the Dirac electrons by measurements of various physical properties under ambient pressure.

New conductors (**89**)_2_[3,3’-Co(1,2-C_2_B_9_H_11_)_2_] and (**89**)_2_[3,3’-Fe(1,2-C_2_B_9_H_11_)_2_] were synthesized based on tetramethyltetraselenafulvalene **89** by anodic oxidation under galvanic conditions in the presence of Na[3,3’-Co(1,2-C_2_B_9_H_11_)_2_] and (Me_3_NH)[3,3’-Fe(1,2-C_2_B_9_H_11_)_2_] [106,107].

Semiconductors (**89**)_3_[Re_2_Cl_8_]_2_CH_3_CN and (**89**)_5_[Re_2_Cl_8_]_2_·6CH_2_Cl_2_ were obtained by electrochemical oxidation at room temperature in the presence of (*n*-Bu_4_N)_2_[Re_2_Cl_8_] in acetonitrile or dichloromethane [108], also known are superconductors such as (**89**)_2_PF_6_ and (**89**)_2_CIO_4_ [109,110,111] and the organic charge-transfer complex (**89**)TCNQ, which is an intrinsic semiconductor [112,113].

The compound **89** and trifluoromethyltetracyanoquinodimethane (CF_3_TCNQ) form two types of charge transfer complexes, (**89**)_2_(CF_3_TCNQ) and (**89**)(CF_3_TCNQ)(PhCl)_1/2_ (from chlorobenzene solution). The first one is semiconductor and the second is a strong donor, i.e., it exhibited metallic properties [114].

Tetramethyltetraselenafulvalene **89** forms radical cationic salts with weakly coordinating anions such as tetrakis(3,5-trifluoromethylphenyl)borate (BArF), dodecamethylcarborane (Me_12_CAR), and hexabromocarborane (Br_6_CAR, which showed a high tendency to *π*-dimerization. The radical cationic salt (**89**)(Me_12_CAR) was obtained by mixing a solution of dodecamethylcarborane in pentane and a solution of selenafulvalene **89** in dichloromethane in a 1:1 molar ratio. Radical-cationic salts (**89**)(BArF), (**89**)(BArF)(CH_2_Cl_2_)_1/2_ and (**89**)(Br_6_CAR) were obtained by mixing a solution of selenafulvalene **89** in dichloromethane with tris(*p*-bromophenyl)aminium cation-radical of tetrakis(3,5-trifluoromethylphenyl)borate or hexabromocarborane taken as salts. The obtained radical-cationic salts are promising for the use in molecular rotors and switches, in which molecular motion is associated with *π*-bonding between opposite parts and depends on the strength of such interactions [115].

The compound **89** is also used in multisensor matrices for the detection of analytes in the gas or liquid phase, which includes, along with tetraselenafulvalene **89**, tetrahalogenated tetraselenafulvalene and other tetrachalcogenafulvalene derivatives that can individually change their physicochemical properties when exposed to analytes or to mixtures of analytes, and these changes can be detected by a sensor or by a set of sensors [116].

Based on bis(ethylenedithio)tetraselenafulvalene **122b** and (AsPh_4_)_2_(Cu_2_Cl_6_), the *κ*-(**122b**)_8_(Cu_2_Cl_6_)(CuCl_4_) and *θ*-(**122b**)_2_(CuCl_2_) salts were obtained by diffusion electrocrystallization of solutions in a mixture of chlorobenzene-ethanol solvents. The obtained salts *κ*-(**122b**)_8_(Cu_2_Cl_6_)(CuCl_4_) and *θ*-(**122b**)_2_(CuCl_2_) exhibited metal-like behavior down to 40 K and 4 K, respectively, and the salt (**122b**)_2_(CuCl_4_) salt showed dielectric properties [117].

The (**122b**)_3_[Cu_2_(C_2_O_4_)_3_](CH_3_OH)_2_ salt, which was obtained by electrochemical oxidation of neutral bis(ethylenedithio)tetraselenafulvalene **122b** in the presence of [(C_2_H_5_)_3_NH]Cu_2_(C_2_O_4_)_2_ in a solution of a mixture of solvents chlorobenzene-methanol, demonstrated antiferromagnetic properties [118].

The lead-containing salts, (**122b**)PbBr_3_ and (**122b**)_2_Pb_2_Br_5_, exhibited metallic resistivity at low temperature [119]. These salts were prepared by electrochemical galvanostatic oxidation of bis(ethylenedithio)tetraselenafulvalene **122b** in the presence of Bu_4_NPbBr_3_ or [Bu_4_NPbBr_3_ + Bu_4_NBr].

Electrocrystallization of compound **122b** gave *θ*-(**122b**)_4_[Fe(CN)_5_NO], (**122b**)_2_[RuBr_5_NO] and (**122b**)_2_[RuCl_5_NO] salts using the nitroprusside anion or its corresponding ruthenium halides ([Fe(CN)_5_NO]^2−^ or [RuX_5_NO]^2−^, where X = Cl or Br) and auxiliary electrolytes as a mixture of solvents (1,1,2-trichlorethylene/ethanol (10 vol%), nitrobenzene/1,2-dichloroethylene (40 vol%)/ethanol (10 vol%) and benzonitrile/ethanol (10%), respectively). The *θ*-(**122b**)_4_[Fe(CN)_5_NO] salt exhibited metallic properties down to 40 K, the (**122b**)_2_[RuBr_5_NO] salt behaved like a semiconductor, and the (**122b**)_2_[RuCl_5_NO] salt was an insulator [120].

Based on the Keggin polyoxometalate ([SMo_12_O_40_]*^n^*^−^) and compound **122b**, the semiconductor (**122b**)_8_[SMo_12_O_40_]_3_·10H_2_O, was obtained, which shows increased conductivity at elevated pressure [121]. The bimetallic oxolate complex (**122b**)_3_[MnCr(C_2_O_4_)_3_]·(CH_2_Cl_2_) exhibited the hybrid properties of a ferromagnet at temperatures below 5.3 K and metal-like conductivity at ambient temperature [122]. Obtained by electrocrystallization, two-dimensional organic metals (**122b**)_4_MBr_4_(PhBr) (M = Cd, Hg) with differently oriented conducting layers in the plane of the conducting layer demonstrated metallic properties, and across the layers-semiconductor behavior, which depends on temperature [123]. Also known are superconductors such as *κ*-(**122b**)_2_TlCl_4_ [124], *κ*-(**122b**)_4_Hg_2.84_Br_8_ [125], *λ*-(**122b**)_2_Fe*_x_*Ga_1-*x*_Cl_4_ (*x* = 0.45) [126], and *λ*-(**122b**)_2_FeCl_4-*x*_Br*_x_* (*x* = 0.4, 0,5 and 0.7) [127] and organic metals such as *κ*-(**122b**)_4_Hg_3_Cl_8_) [125], *θ*-(**122b**)_4_Ni(CN)_4_ [128], and *κ*-(**122b**)_2_Mn[N(CN)_2_]_3_ [129].

Dimethyl(ethylenedioxy)tetraselenafulvalene **194** was synthesized by the cross-coupling of 4,5-dimethyl-1,3-diselenole-2-selone and 4,5-ethylenedioxy-1,3-diselenole-2-selone [130]. The superconductors *κ*-(**194**)_2_[Au(CN)_4_](solv.) (solv. = 1,3-dioxolane, 2,5-dihydrofuran, tetrahydropyran, 1,3-dioxane, 3,4-dihydro-2*H*-pyran or 1,4-dioxane) [130,131] and radical cationic salts of the general formula (**194**)_2_X (X = PF_6_, AsF_6_, SbF_6_) with metallic properties [132] were obtained based on compound **194**.

**Scheme 56:**
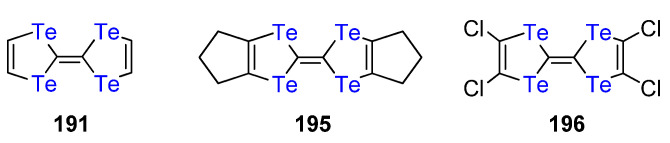
Well-known tetratellurafulvalenes, which are effective as electron-donor compounds and used for the preparation of conductive materials.

The well-known tellurium-containing fulvalenes effective as *π*-donors are tetratellurafulvalene **191**, hexamethylenetetratellurafulvalene **195**, and tetrachlorotetratellurafulvalene **196** (Scheme 56).

Tetratellurafulvalene **191** in the form of a charge transfer complex with tetracyanoquinodimethane is used in the production of modified electrically conductive thin graphene films [133].

Tetratellurafulvalene **191** and hexamethylenetetratellurafulvalene **195** are used in photoelectric conversion functional devices as substrates in the form of charge transfer complexes that exhibit near-IR absorption [134]. In addition, fulvalenes **191**, **195,** and tetramethylthiotetratellurafulvalene are used in electroluminescent devices as electron carriers and electron donors. Such organic electroluminescent devices show low operating voltage and high luminescence intensity due to the low resistance of the organic layer [135].

Based on hexamethylenetetratellurafulvalene **195**, single-crystal complexes with 2,5-diethyltetracyanoquinodimethane (Et_2_TCNQ) and bis-1,2,5-thiadiazolotetracyanoquinodimethane (BTDA-TCNQ), which have a packing structure with alternately stacked donor-acceptor molecules, were prepared. These two complexes showed high conductivity despite their disadvantageous packing method, which usually leads to materials with dielectric properties [136].

The complex of fulvalene **195** with 2,5-diethyltetracyanoquinodimethane, (**195**)(Et_2_TCNQ)(THF)_x_ (x = 0.5–1), is metallic at temperatures above 200 K [137], while its complex with trifluoromethyltetracyanoquinodimethane (**195**)_4_(CF_3_TCNQ)_3_ is a semiconductor [114].

Tetrachlorotetratellurafulvalene **196** is used in multisensor matrices for the detection of analytes in the gas or liquid phase [116].

## 5. Conclusions

The derivatives of 1,4-diselenafulvene and 1,4,5,8-tetraselenafulvalene and their tellurium analogs are important scaffolds and valuable heterocyclic building blocks for organic synthesis and donor units for the preparation of charge-transfer complexes and radical ion salts. The 1,4,5,8-tetraselenafulvalene derivatives and its tellurium analogs have shown themselves to be useful electron donors for the preparation of various conducting materials including Dirac electronic systems [31,32,53,54,55,56,57], superconductors [4,5,6,8,9,10,11,15,16,17,18,19,20,21,22,24,47,75,78,85,106,107,109,110,111,124,125,126,127], semiconductors [52,67,71,73,75,108,112,113,114,120,121,123] and compounds with ferromagnetic properties [7,15,23,48,76,114,118,122]. Well-known tetraselena- and tetratellurafulvalenes, which are effective as electron-donor compounds and most commonly used for the preparation of conductive materials, are outlined in Scheme 55 and Scheme 56.

Regarding the synthesis of 1,4,5,8-tetraselenafulvalene and its derivatives, the efficient approaches based on cross-coupling reactions of 1,3-selenole-2-one, 1,3-selenole-2-thione and 1,3-selenole-2-selone, which usually proceed in the presence of triethylphosphite, have frequently been used. These methods have been successfully developed and improved.

However, some efficient methods deserve to be mentioned. The method for preparation of diselenafulvene **1** and tetraselenafulvalene **2** based on selenium and sodium acetylide is a convenient and practical synthetic approach (Scheme 29) [78]. This approach, as well as the method depicted in Scheme 1 [34], have the advantages of using cheap, non-toxic selenium powder as the selenium source and commercially available acetylene or sodium acetylide.

Very important is a general synthetic approach to a series of alkylenedithio- **121a**–**c** and bis(alkylenedithio)tetraselenafulvalenes **122a**–**c** (Scheme 38) [88]. Tetraselenafulvalenes **122a**–**c** (especially compound **122b**, Scheme 55) have frequently been used for preparation of various conducting materials including organic metals and superconductors [117,118,119,120,121,122,123,124,125,126,127,128,129].

The efficient approach to a number of polycyclic selenium-containing tetrathiafulvalene vinylogues of the dendralene type **155a**–**c**, **156a**–**d**, **157**, and **158** bearing a 1,3-diselenole moiety using Vilsmeyer–Haack and Wittig–Horner reactions is worth mentioning (Scheme 46) [97].

A valuable synthesis of tetratellurafulvalene **191** in 26% yield by the optimized procedure with the use of tetrabromoethene at the final stage of cyclization is also very important (Scheme 54) [105]. This approach makes it possible to reliably obtain sufficient amounts of compound **191**, which can be used for investigation of its potential as a building block for the preparation of functionalized electron donor compounds and the preparation of novel radical cation complexes and charge-transfer salts based on tetratellurafulvalene [105].

Obviously, the possibilities of 1,4-diselenafulvenes, 1,4,5,8-tetraselenafulvalenes, and their tellurium analogs for use in organic synthesis and in the preparation of conductive materials are far from being exhausted. Further research will lead to the synthesis of previously unknown complex compounds and preparation of novel conductive materials with new useful combinations of physicochemical properties.

## Data Availability

Not applicable.
